# Coronary artery volume to left ventricular myocardial mass ratio in detecting primary microvascular angina: a retrospective observational study in Chinese population

**DOI:** 10.1186/s12872-025-04978-0

**Published:** 2025-07-28

**Authors:** Linlin Sun, Yanli Yu, Xincheng Li, Guoxin Tong, Beibei Gao, Haipeng Liu, Zhen Wang

**Affiliations:** 1 Zhejiang Chinese Medical University, Hangzhou, China; 2 Affiliated Hangzhou First People’s Hospital, Westlake University School of Medicine, Hangzhou, China; 3 Centre for Intelligent Healthcare, Coventry University, Coventry, CV1 5RW UK

**Keywords:** Coronary computed tomography angiography, Coronary artery volume to left ventricular myocardial mass ratio, Primary microvascular angina, Fractional flow reserve, Coronary microvascular dysfunction

## Abstract

**Background:**

The ratio of coronary artery volume to left ventricular myocardial mass (V/M) reflects the hemodynamic association between epicardial coronary arteries and myocardium. Evidence suggests a significant decrease in V/M among patients with primary microvascular angina (PMVA). However, V/M values may be influenced by anatomic and physiological varieties, necessitating independent evaluation in different ethnic groups. We aimed to explore the potential of V/M in predicting PMVA in Chinese population.

**Methods:**

We conducted a retrospective case-control analysis on 23 PMVA patients and 25 controls matched by age, sex, body mass index, smoking history, hypertension, diabetes, and dyslipidemia. For each patient, the computed tomography (CT) images were three-dimensionally reconstructed to calculate patient-specific V/M and vessel-specific V/M, as well as CT-derived fractional flow reserve (CT-FFR). The results in both groups were compared using t-test or Mann-Whitney U test.

**Results:**

Compared to the control group, the PMVA group had significantly higher total myocardial mass and lower average V/M (*P* < 0.05 for both). Regarding vessel-specific V/M, PMVA group had significantly lower values of left anterior descending artery and left circumflex artery (*P* < 0.05 for both), with no significant inter-group difference in right coronary artery (*P* > 0.05). There was no significant difference between two groups in vessel-specific CT-FFR (*P* > 0.05 for all).

**Conclusion:**

The abnormally decreased V/M value may serve as a potential biomarker of PMVA in Chinese population.

## Introduction

Angina pectoris is a typical symptom of coronary artery disease (CAD) that affects approximately 112 million people globally. Up to 70% of angina patients undergoing invasive coronary angiography were found not to have obstructive CAD (defined as narrowing in lumen diameter ≥ 50% on coronary angiography) [1, 2], where coronary microvascular dysfunction (CMD) is a main pathology. Coronary microvasculature is characterized by vessels with diameter less than 0.5 mm, constituting 90% of the total myocardial blood volume and flow resistance in coronary circulation [3]. These coronary microvessels form the primary sites for myocardial metabolism, supplying blood, oxygen, and participating in the clearance of metabolic by-products [4]. CMD without concomitant obstructive CAD is termed primary microvascular angina (PMVA), characterized by impaired coronary flow reserve (CFR) and diffuse vasomotor dysfunction without apparent epicardial coronary artery obstruction [5].

Diagnosing PMVA often requires invasive procedures [6]. Primary microvascular angina (PMVA), characterized by impaired and diffuse vasomotor dysfunction without epicardial obstruction, presents significant diagnostic challenges due to its reliance on invasive procedures. While fractional flow reserve (FFR) and index of microcirculatory resistance (IMR) are gold standards for assessing ischemia and CMD, respectively, both have limitations. FFR primarily evaluates epicardial vessels and is influenced by microvascular dysfunction [7], whereas IMR focuses on microvascular evaluation [4, 8], but the procedure is highly invasive and lacks assessment of myocardial function. CFR, though commonly used, reflects combined macrovascular and microvascular hemodynamics, making it less specific for CMD diagnosis [9]. These limitations highlight the need for non-invasive and comprehensive diagnostic approaches for PMVA.

The ratio of coronary artery volume to myocardial mass (V/M) has been proposed as a quantitative indicator of potential imbalance between coronary blood supply and myocardial demand [10]. Low V/M is an independent predictor of ischemia and may serve as a potential indicator of CMD [11]. Compared to CFR and IMR, V/M can be non-invasively derived from clinical imaging. Coronary computed tomography angiography (CCTA) is a common non-invasive examination that provides the anatomic details of coronary arteries and myocardium. CCTA images can be three-dimensionally reconstructed to estimate the coronary artery volume and the mass of the left ventricle, thereby obtaining the V/M ratio. In addition, CCTA-reconstructed three-dimensional geometry of coronary arteries can be used for computational fluid dynamics simulation of patient-specific hemodynamic parameters (e.g., flow rate and FFR) to assess the risk of myocardial ischemia in CMD patients [12, 13].

There are ethnic differences in the incidence, severity, and outcomes of CAD, as well as cardiac anatomy [14]. In a multiethnic study, V/M was found to be more significantly elevated in suspected CAD patients of East Asian descent compared with those from Caucasian and South Asian descents [15]. It was found that V/M might be associated with PMVA in the mestizo population of British Columbia, the evaluation in different ethnic groups is warranted [11].

However, as far as we know, there is a lack of comprehensive assessment on the diagnostic value of V/M for PVMA in East Asian population.

To fill this research gap, we observed the V/M values in a Chinese cohort of PMVA patients. We hypothesize that the V/M ratio can serve as an effective, non-invasive marker to predict PMVA in the Chinese population. The results provided new evidence on the diagnostic value of V/M for PMVA in a regional and ethnic context.

## Methods

### Study design

**Fig. 1:**
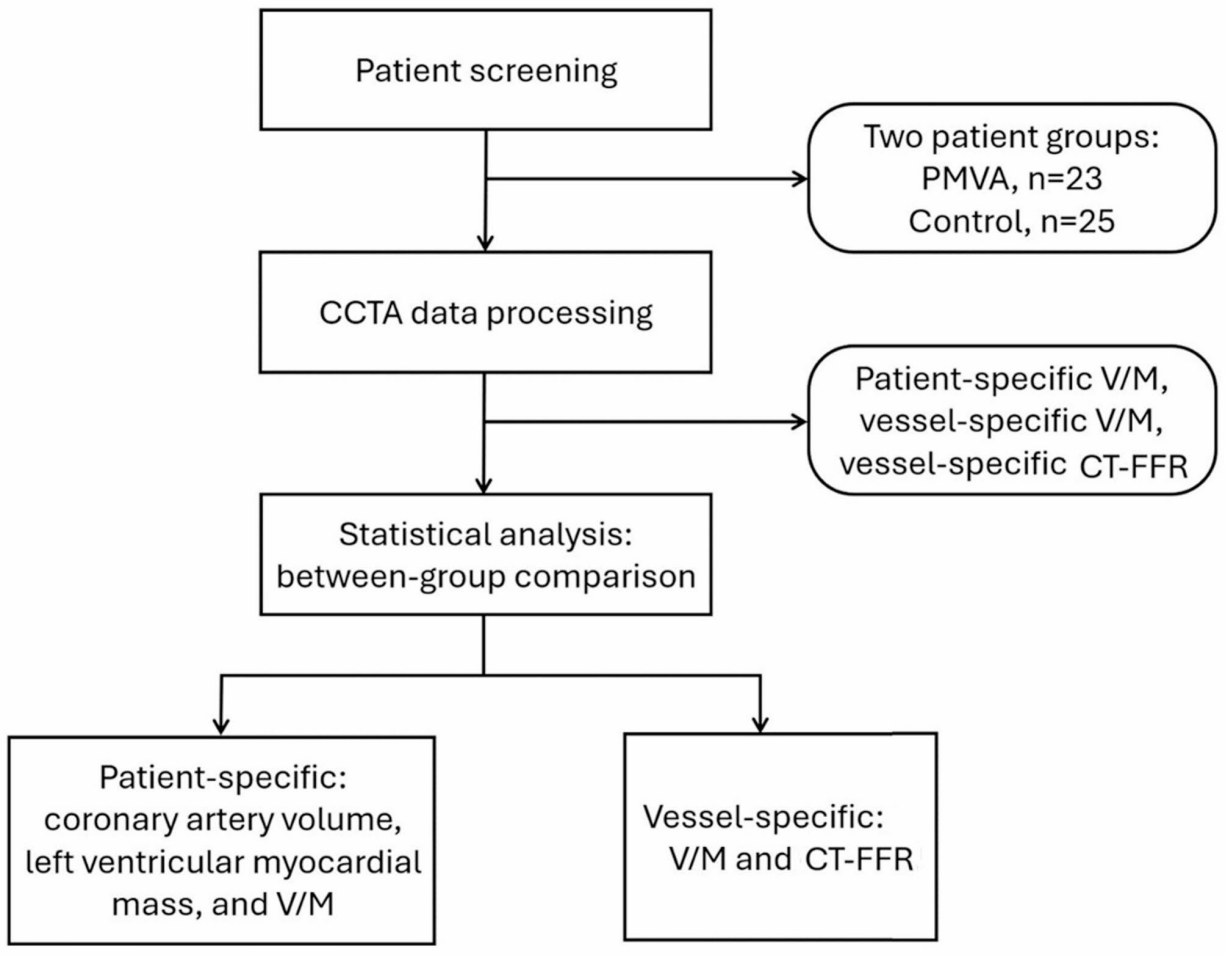
Workflow diagram of this study. CCTA: coronary computed tomography (CT) angiography, PMVA: primary microvascular angina, V/M: the ratio of coronary artery volume to left ventricular myocardial mass, CT-FFR: CT-derived fractional flow reserve

This study was a retrospective case-control analysis based on matched PMVA and control groups. PMVA patients were identified and collected based on medical records and diagnostic criteria. Control group was assembled to match the PMVA patients based on demographic and clinical characteristics. The two groups were compared in patient-specific V/M and vessel-specific V/M as well as CT-FFR values (Fig. 1).

The PMVA group included patients diagnosed with PMVA at the Hangzhou First People’s Hospital in China from August 2021 to August 2023. Two experienced cardiologists independently evaluated each patient based on the PMVA diagnostic criteria proposed by the Coronary Vasomotion Disorders International Study Group (COVADIS) [16]. In case of any inconsistent assessment conclusions, a third physician was introduced as an arbitrator. The control group comprised patients who underwent CCTA for angina during the same period and were not diagnosed with CMD.

#### Inclusion criteria

Patients that had CCTA scanning and IMR measurement and diagnosed as PMVA or without CMD.

#### Diagnostic criteria for PMVA

1. Symptomatic requirements
  Presence of symptoms suggestive of myocardial ischemia: Typical symptoms include chest discomfort (e.g., exertional angina, rest angina, or atypical angina).
  Exclusion: Isolated dyspnea without angina.
2. Objective evidence of myocardial ischemia
  Demonstrated through one or more of the following modalities:
  Stress echocardiography,
  myocardial perfusion imaging (MPI),
  cardiac magnetic resonance imaging (CMR) with stress perfusion,
  positron emission tomography (PET).
3. Absence of obstructive coronary artery disease (CAD)
  Defined as <50% luminal diameter reduction on coronary angiography and FFR values≥0.75.
4. Evidence of microvascular dysfunction
  Index of IMR ≥25 or CFR ≤2.0.

#### Exclusion criteria

1. Allergy to contrast agents: Includes documented hypersensitivity to iodinated or gadolinium-based contrast media required for diagnostic imaging.
2. Special populations unsuitable for examination:
  Pregnant women: Potential teratogenic effects of pharmacological stress agents or contrast agents.
  Lactating women: Risk of transmission of contrast agents or medications to the infant through breast milk.
3. History of invasive cardiovascular interventions or systemic therapy:
  Coronary stent placement or coronary artery bypass grafting (CABG).
  Ongoing systemic therapies that could affect cardiovascular function, including immunosuppressive agents or targeted cancer therapies.
4. Severe cardiac conditions:
  Arrhythmias: Persistent atrial fibrillation with rapid ventricular response, ventricular tachycardia, or complete atrioventricular block.
  Decompensated heart failure: NYHA Class III-IV or left ventricular ejection fraction (LVEF) <40%.
5. Severe obesity: Body mass index (BMI) >35 kg/m², which may compromise imaging quality or procedural safety.
6. Severe hepatic or renal dysfunction:
  Hepatic dysfunction: Alanine aminotransferase (ALT) or aspartate aminotransferase (AST) >3 times the upper limit of normal.
  Renal dysfunction: Estimated glomerular filtration rate (eGFR) <30 mL/min/1.73 m² or end-stage renal disease requiring dialysis.
7. Systemic or immune-related diseases:
  Autoimmune disorders: Active systemic lupus erythematosus, rheumatoid arthritis, or similar conditions.
  Malignant tumors: Active or recently treated malignancies with cardiovascular involvement.
8. Inability to comply with trial requirements:
  Cognitive impairments or lack of consent due to neurological or psychological conditions.
  Geographical or logistical barriers that prevent adherence to follow-up protocols.

**Fig. 2:**
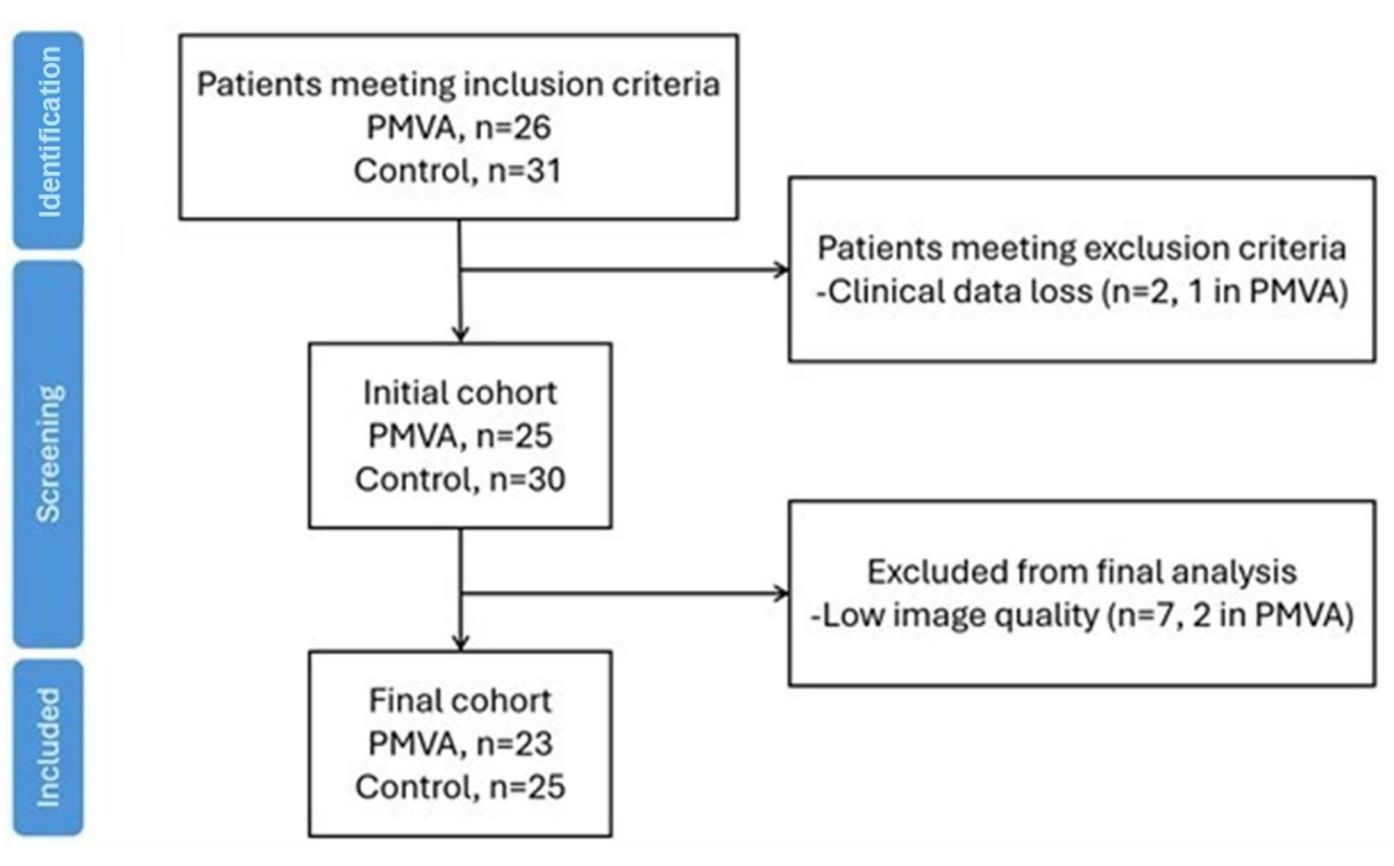
Patient screening and matching process

The imaging data of initially selected subjects were reviewed, with seven patients further exclude for low image quality (Fig. 2). In the finally included cohort, there were 23 subjects in the PMVA group and 25 subjects in the control group. It is noteworthy that the patients in the control group were symptomatic for angina, of which 11 were diagnosed with CAD.

### CCTA acquisition

The CCTA examination was performed using a Siemens dual-source 128-row computed tomography (CT) scanner, with the scanning range from 1.0 cm below the tracheal bifurcation to 1.5 cm below the cardiac diaphragm level/heart lower margin.

#### Scanning parameters

Dual Vectron tube, rotation speed of 0.25 s/rotation; collimator width of 2 mm × 96 mm × 0.6 mm; highest temporal resolution of 66 ms; flying focal spot technology: 0.4 mm × 0.5 mm. Patients were trained to hold their breath in advance, and their heart rate was controlled to be below 75 beats/min. If the resting heart rate of the patient was > 75 beats/min, metoprolol tablets were administered orally before the examination at a dose of 25–75 mg to achieve the standard heart rate before starting the examination. All patients did not receive nitroglycerin during the examination. Scans for all patients were completed within one cardiac cycle using prospective electrocardiogram gating technology. The optimal phase (usually late diastole) was selected for image reconstruction on the post-processing workstation (Siemens Syngo.via).

### Calculation of patient-specific V/M, vessel-specific V/M and vessel-specific CT-FFR

**Fig. 3:**
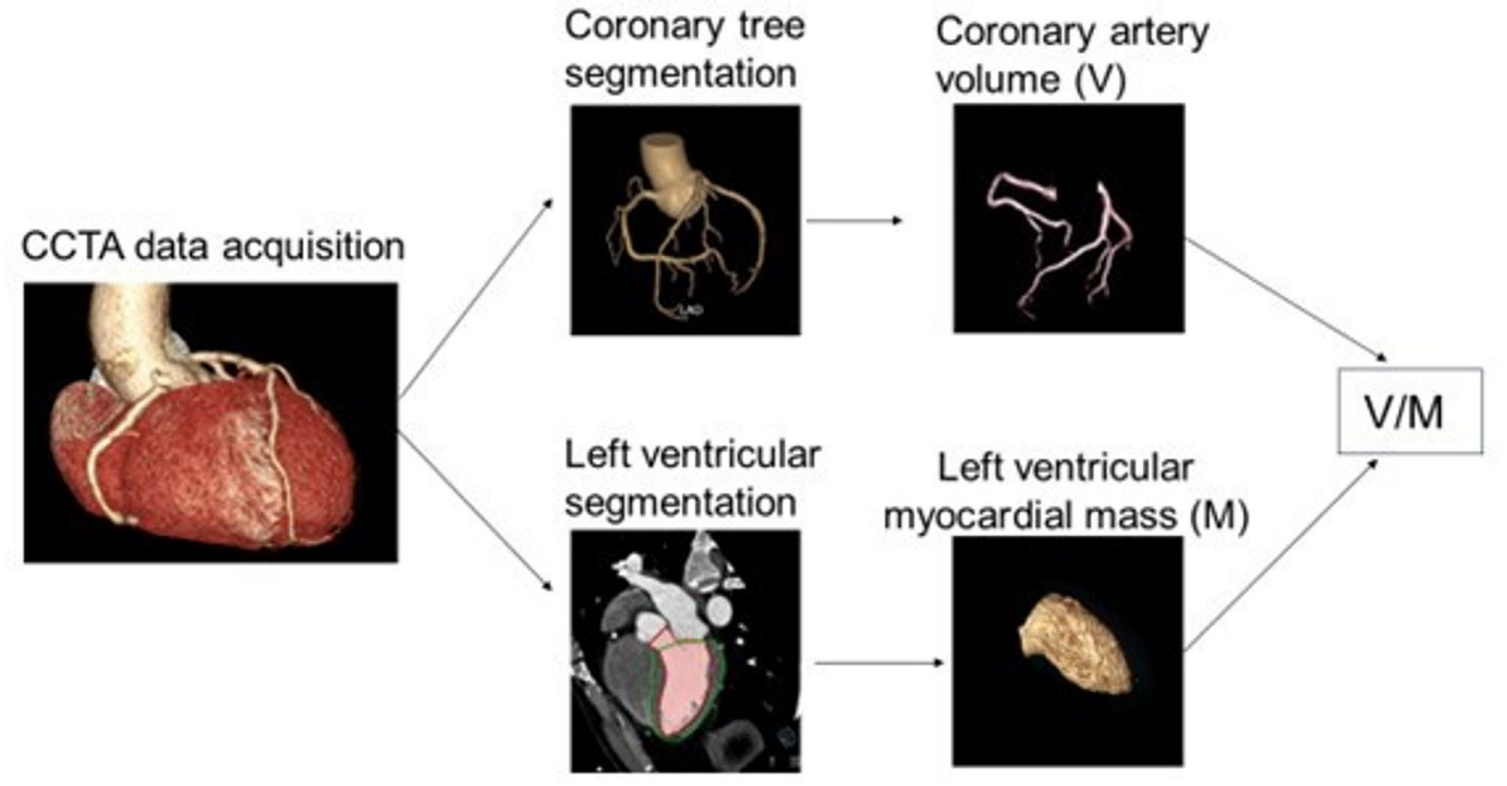
Schematic diagram of V/M calculation method. CCTA: coronary computed tomography (CT) angiography, V/M: the ratio of coronary artery volume to left ventricular myocardial mass

The V/M measurement was performed on PHIgo research platform (GE Pharmaceutical Co., Ltd., Massachusetts, United States). Figure 3 illustrates the workflow of V/M measurement. CCTA images were automatically segmented to obtain the regions of interest of coronary artery and left ventricular myocardium.

#### Patient-specific V/M calculation

For each patient, the total coronary artery volume (V) and left ventricular myocardial volume were determined from the three dimensionally reconstructed structures of the regions of interest. The left ventricular myocardial volume was multiplied by the average density of myocardial tissue (1.05 g/ml) to calculate the left ventricular myocardial mass (M). Finally, the patient-specific V/M ratio was derived.

#### Vessel-specific V/M calculation

**Fig. 4:**
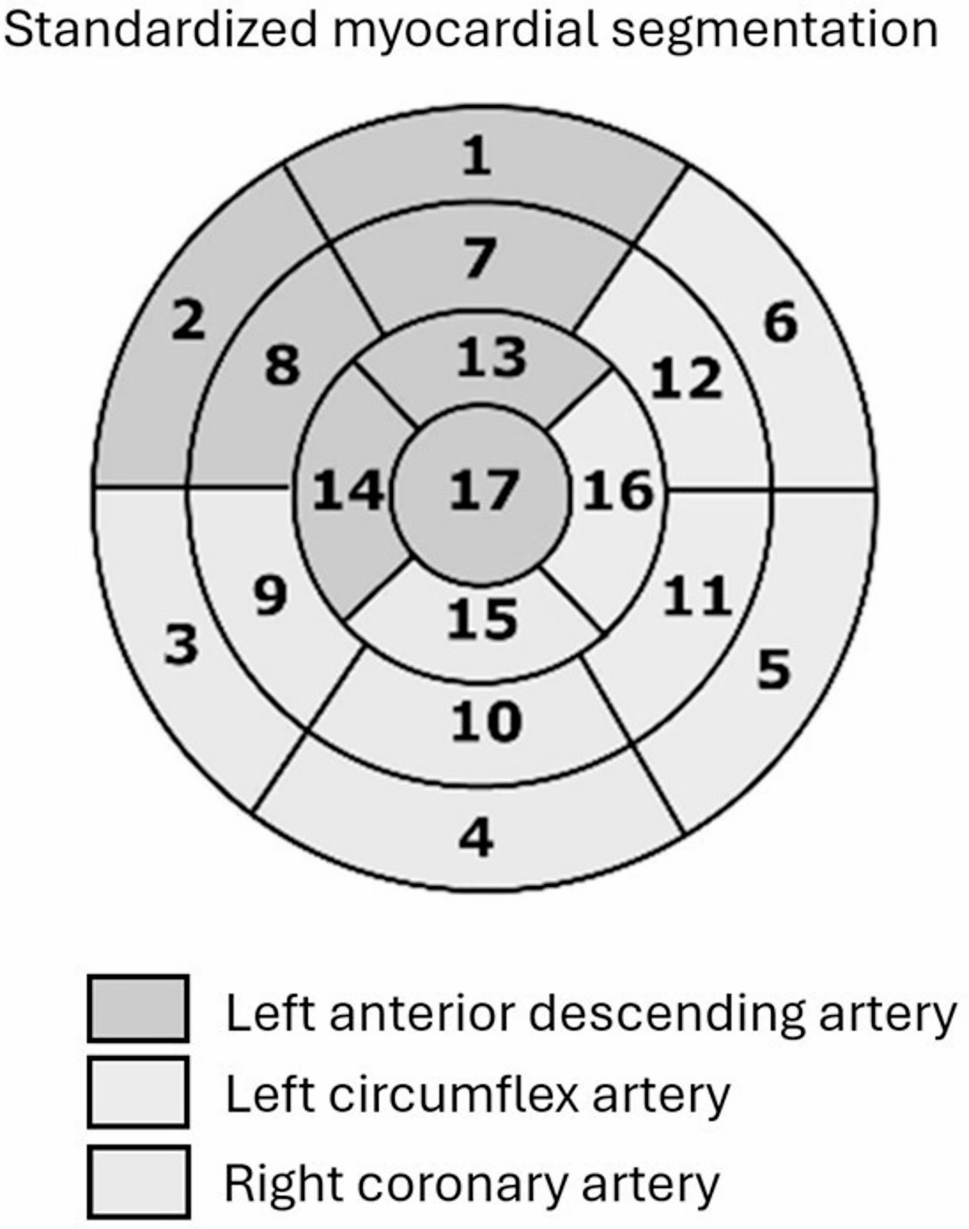
Standardized myocardial segmentation proposed by American Heart Association

The left anterior descending artery (LAD), left circumflex artery (LCX), and right coronary artery (RCA) are the main coronary artery vessels. On microcirculatory level, the three main vessels supplies blood to specific myocardial territories [17–19]. Therefore, we calculated vessel-specific V/M using the PHIgo research platform. The coronary artery tree was segmented to obtain the vascular volumes of the three epicardial arteries (V_LAD_, V_LCX_, and V_RCA_). The CQK software of PHIgo automatically obtained the American Heart Association 17-segment (AHA) perfusion areas (Fig. 4), with myocardial segments 1, 2, 7, 8, 13, 14, and 17 defined as the region supplied by LAD, myocardial segments 5, 6, 11, 12, and 16 defined as the region supplied by LCX, and myocardial segments 3, 4, 9, 10, and 15 defined as the region supplied by RCA [20]. The myocardial mass of the three regions was then calculated. Finally, the vessel-specific V/M ratio was calculated by dividing the coronary artery volume of each epicardial coronary artery by the corresponding myocardial mass. Some outliners, especially in some right dominant cases, were excluded.

#### Vessel-specific CT-FFR analysis calculation

For each coronary artery vessel, CT-FFR analysis based on deep learning was performed using the DEEPVESSEL FFR software (Koya Medical Technology Co., Ltd., Beijing, China). DEEPVESSEL FFR software automatically measures the severity of coronary artery stenosis and calculates FFR of each vessel as translesional pressure ratio, i.e., the ratio between pressure values distal and proximal to a stenosis. A unique deep bidirectional long-term recurrent neural network (DBL-RNN) algorithm was used to rapidly calculate the CT-FFR values.

### Statistical analysis

Data analysis was conducted using SPSS software (Version 28.0, IBM Corp.; Armonk, NY, United States). The Pearson’s chi-square test was employed for analyzing categorical data, and results were reported as frequencies and percentages. Normality and homogeneity of variance for continuous data were initially assessed. Normally distributed continuous data were described as mean ± standard deviation and compared between groups using independent sample t-tests. Non-normally distributed data were described as median (P25, P75) and compared using the Mann-Whitney U test. Violin plots were employed for between-group comparisons and vessel-specific V/M analysis, and receiver operating characteristic (ROC) curves were utilized to assess the ability of V/M to identify patients with PMVA.

## Results

### General data

Comparison of general clinical data between the two groups is presented in Table 1. No significant difference was observed between the PMVA and control groups in basic clinical information or blood biomarkers (*P* > 0.05 for all).

**Table 1:** Comparison of general clinical data between the two groups

	Control	PMVA		t/z/ χ² value	*P* value
Age (year)	64.92±8.34	62.48±10.19		0.912	0.367
Men [case (%)]	12 (48)	12 (52)		0.083	0.773
BMI	24.53±3.34	24.08±3.53		0.432	0.668
Smoking [case (%)]	6 (24)	5 (22)		0.035	0.852
Hypertension [case (%)]	14 (56)	15 (65)		0.426	0.514
Diabetes mellitus [case (%)]	0 (0)	2 (9)		2.268	0.132
Dyslipidemia [case (%)]	3 (12)	2 (9)		0.140	0.708
HbA1c [M (P25, P75), mmol/L]	5.6 (5.3,6.4)	5.5 (5.2,5.9)		-0.680	0.496
UREA [M (P25, P75), mmol/L]	5.4 (4.1,7.0)	5.830(4.6,7.5)		-0.819	0.413
eGFR (mean±SD, mmol/L)	73.47±15.21	67.17±20.82		1.187	0.241
LDL-C (mean±SD, mmol/L)	2.26±0.83	2.26±0.78		-0.014	0.989
HDL-C (mean±SD, mmol/L)	1.22±0.33	1.16±0.27		0.695	0.491
UA (mean±SD, mmol/L)	351.92±81.92	333.52±101.50		0.685	0.497
Cr [M (P25, P75), μmmol/L]	79.5 (72.8, 90.0)		84.0 (74.0, 100.0)	-0.501	0.617

*P25 and P75* 25th and 75th percentiles, *SD* Standard deviation, *HbA1c* Glycated hemoglobin, *UREA* Urea, *eGFR *Estimated glomerular filtration rate, *HDL-C* High-density lipoprotein cholesterol, *LDL-C* Low-density lipoprotein cholesterol, *UA* Uric acid, *Cr* Creatinine

### Correlation analysis of FFR and IMR

**Fig. 5:**
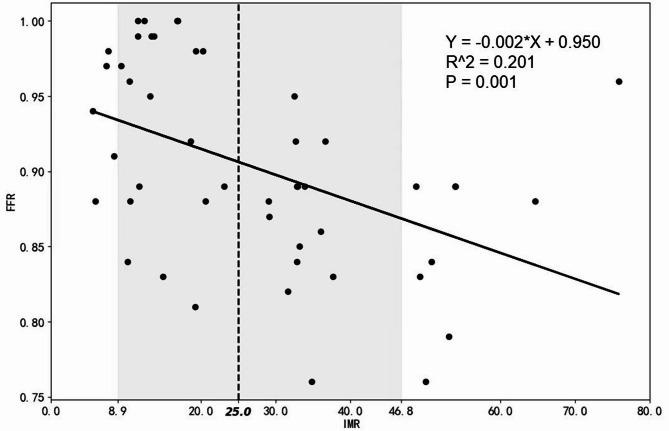
Correlation analysis of FFR and IMR

The relationship between FFR and IMR was analyzed using a scatter plot (Fig. 5). The figure reveals that with the increase in FFR, there is a downward trend in IMR.

### Patient-specific parameters: myocardial mass, coronary artery lumen volume, and V/M ratio

**Fig. 6:**
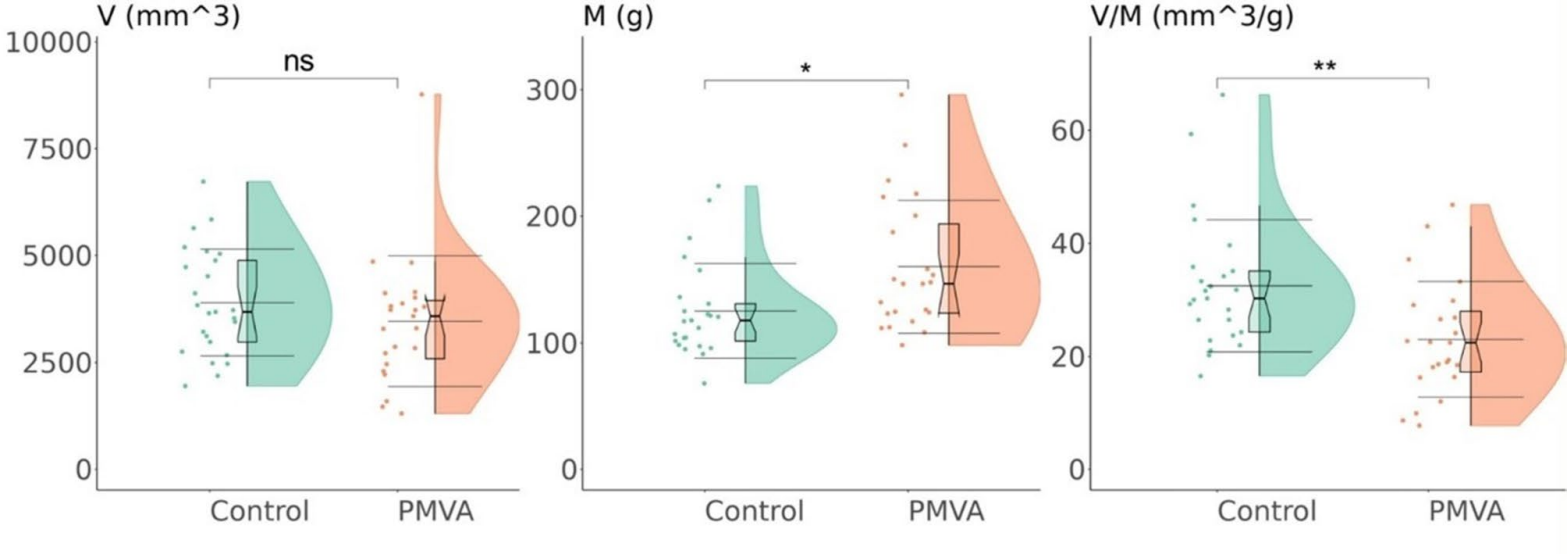
Comparison of coronary artery lumen volume (V), myocardial mass (M), and V/M ratio between the PMVA and control groups. **A** Distribution of coronary artery lumen volume in two groups. **B** Distribution of myocardial mass in two groups. **C** Distribution of V/M ratio in two groups. The internal boxplots depict the median and quartiles, with the bold horizontal line representing the median, and the upper and lower bounds corresponding to the upper and lower quartiles. The three long horizontal lines represent the mean ± standard deviation. “ns” indicates non-significant difference, “*” and “**” indicates significant differences (*P* < 0.05 and *P* < 0.01). PMVA: primary microvascular angina

As shown in Fig. 6, compared with the control group, PMVA group is significantly higher in the total myocardial mass, and lower in the V/M ratio (*P* < 0.05 for both). However, no significant difference in coronary artery lumen volume was observed between the two groups.

**Fig. 7:**
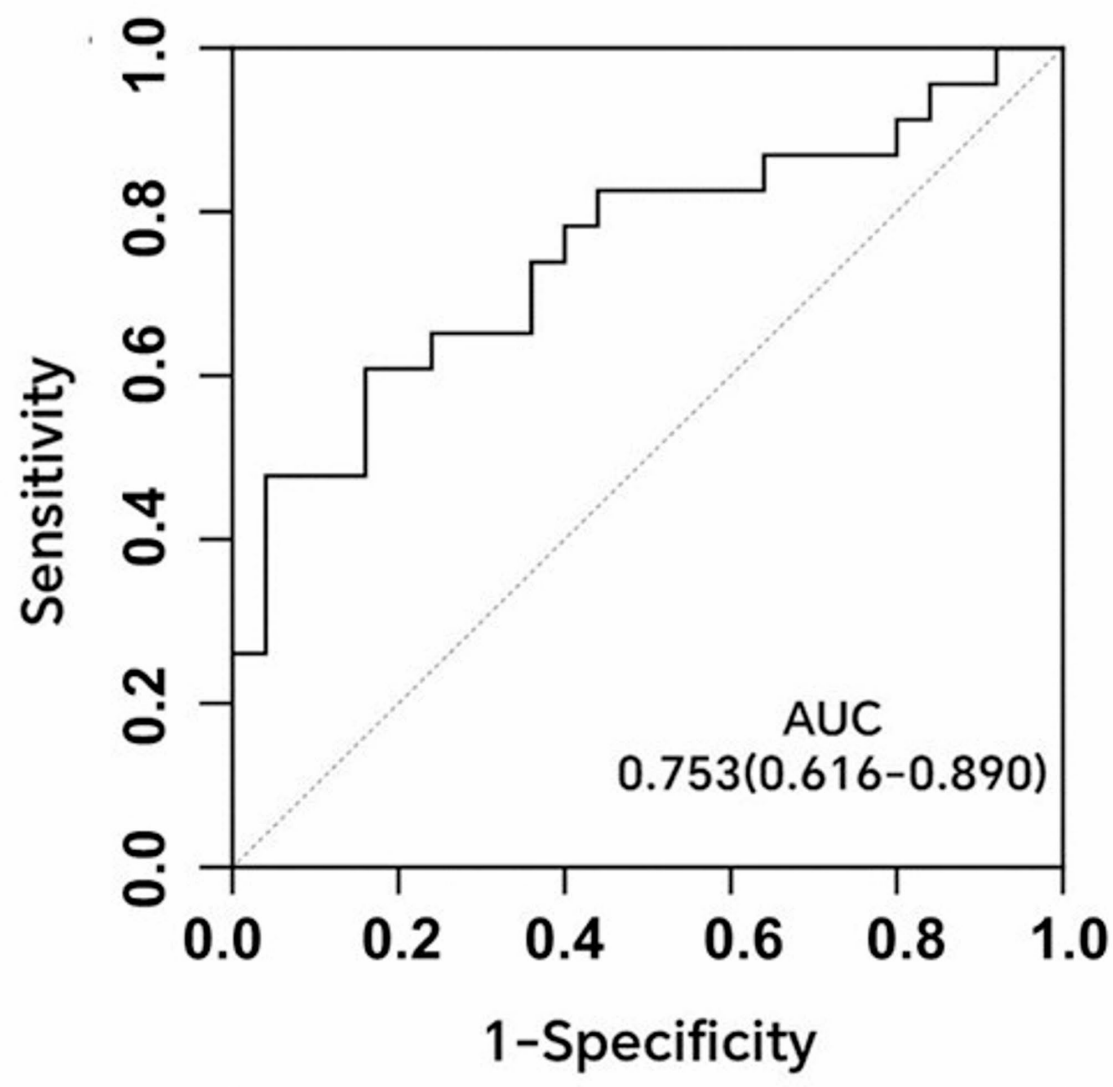
Receiver operating characteristic (ROC) curve of patient-specific V/M. AUC: area under curve

ROC result indicated that patient-specific V/M could effectively predict PMVA, with an area under the curve of 0.753 (95% CI: 0.616–0.890, *P* < 0.01), as depicted in Fig. 7.

### Microvascular functional assessment

All PMVA patients demonstrated impaired vasodilatory capacity with invasive microvascular resistance index (IMR) ≥ 25 (100% sensitivity). Within this group, 5/23 patients (21.7%) exhibited concurrent coronary flow reserve (CFR) ≤ 2.0, indicating more severe ischemia in coronary circulation globally. Control subjects maintained normal physiological ranges for both parameters.

### Vessel-specific parameters: V/M and CT-FFR

**Fig. 8:**
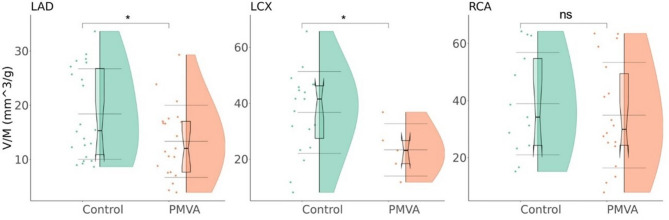
Comparison of the vessel-specific V/M values in left anterior descending artery (LAD), left circumflex artery (LCX), and right coronary artery (RCA) between PMVA and control groups. The internal boxplots depict the median and quartiles, with the bold horizontal line representing the median, and the upper and lower bounds corresponding to the upper and lower quartiles. The three long horizontal lines represent the mean ± standard deviation. “ns” indicates non-significant difference, “*” indicates significant differences (*P* < 0.05)

As Fig. 8 shows, compared to the control group, the PMVA group is significantly lower in vessel-specific V/M values of LAD and LCX (*P* < 0.05 for both), but not RCA (*P* > 0.05).

Regarding the vessel-specific CT-FFR values, there was no significant difference between the PMVA and control groups in LAD (0.88 ± 0.04 vs. 0.87 ± 0.04, *P* > 0.05), LCX (0.93 ± 0.02 vs. 0.91 ± 0.03, *P* > 0.05), or RCA (0.90 ± 0.04 vs. 0.89 ± 0.05, *P* > 0.05).

## Discussion

We observed a significant decrease in patient-specific V/M ratio in patients with PMVA. As far as we know, this is first observation in East Asian population. 21.7% (5/23) of PMVA patients demonstrated concurrent CFR ≤ 2.0, suggesting superimposed functional impairment of adenosine-mediated vasodilation in advanced disease subgroups. In addition, we found a decrease in the vessel-specific V/M of the LCX and LAD in the PMVA group. In comparison, the CT-FFR values were not significantly different between the PMVA and control groups. These results commonly highlight the potential diagnostic value of V/M for PMVA. Notably, the observed V/M reduction was predominantly driven by increased myocardial mass, whereas coronary arterial volume remained comparable between groups. This finding partially contrasts with the mechanistic framework proposed by Grover et al. who attributed V/M reduction to coordinated decreases in coronary volume and myocardial mass secondary to endothelial-dependent vasodilatory dysfunction [11]. These discrepancies in pathophysiological may indicate a dual-pathway model for V/M imbalance: myocardial remodeling predominates in early-stage disease or East Asian cohorts, whereas endothelial dysfunction predominates in advanced stages or Hispanic populations. This pathophysiological pattern exhibits cross-disease congruence with mechanisms underlying major adverse cardiovascular events (MACE) post-transcatheter aortic valve replacement (TAVR). TAVR cohort [21] analyses revealed remodeling-driven pathology, demonstrating significantly elevated left ventricular mass in MACE patients with preserved epicardial coronary volume, collectively reducing V/M ratios. Histological quantification confirmed coronary microvascular rarefaction, directly correlating with impaired microcirculatory reserve [22]. These findings collectively suggest that myocardial remodeling coupled with impaired microvascular adaptation constitutes a convergent terminal pathway for diverse cardiovascular pathologies.

Our data demonstrate a significant inverse correlation between FFR and IMR (Fig. 5). Elevated IMR reduces translesional pressure gradients (ΔP = Pa − Pd) by limiting maximal coronary blood flow (Qmax) [23], which would theoretically increase FFR due the reduced trans-stenotic pressure drop. However, this anticipated outcome may be counteracted by the microvascular dependency of adenosine response. In patients with microvascular dysfunction, adenosine induces paradoxical hyperemia—termed “hyperemic overshoot" [24]—characterized by excessive dilation of residual vascular beds. This aberrant response may exacerbate the decline in distal pressure (Pd), ultimately reducing FFR. Van de Hoef et al. posited that the interplay between microvascular resistance and epicardial stenosis is a critical determinant of FFR heterogeneity [25]. Our findings corroborate this viewpoint and suggest that IMR may serve as a correction factor to refine FFR interpretation. Specifically, the inverse FFR–IMR relationship (Fig. 5) reflects the dynamic interaction between microvascular dysfunction (e.g., capillary rarefaction ≤ 300/mm²) and macrovascular pathophysiology (e.g., diffuse atherosclerosis). These results emphasize the necessity of integrating IMR assessment into FFR evaluation.

Structural microcirculatory changes and functional arteriolar dysregulation are two major pathological mechanisms of CMD and can coexist [2]. The structural microcirculatory changes can decrease myocardial blood flow and/or impair maximal microvascular vasodilation capacity, resulting in myocardial ischemia. To mitigate the ischemia, the heart may undergo remodeling characterized by myocardial hypertrophy and alterations in ventricular wall stress [26], ultimately leading to an augmented myocardial mass. Accordingly, we observed a greater myocardial mass in the PMVA group compared to the control group. In addition, structural changes may lead to increased sensitivity of smooth muscle cells to vasoconstrictor stimuli (such as endothelin-1, acetylcholine, serotonin) [1]. Functional arteriolar dysregulation results from endothelial dysfunction, reduced flow-mediated dilation, decreased response to increased shear stress of nitric oxide, and impaired relaxation of smooth muscle cells, thereby influencing coronary artery luminal volume. Thus, V/M ratio reflects both functional and structural changes as a result of many cardiovascular risk factors (e.g., smoking status) [27]. V/M ratio may aid in revealing the driving factors behind hypertensive pressure loss and angina pectoris in the absence of obstructive CAD [28].

The PMVA and control groups were not significantly different in basic clinical information, enabling us to focus on V/M ratio without involving traditional risk factors of CMD. Coronary microvascular function declines with age and is associated with factors such as smoking [29], advanced age [30], obesity [31], hypertension [32], dyslipidemia [33], and hyperglycemia [34]. While clinical presentations in patients with CMD are similar between males and females, studies have consistently shown an increase in prevalence of CMD among females, especially postmenopausal women [35], suggesting the involvement of other yet undetermined factors in the occurrence and progression of CMD [36–38].

Patients with PMVA secondary to CMD may present with typical angina, atypical symptoms, or angina-equivalent symptoms, often manifesting as exertional retrosternal pressure, chest pain, or discomfort and/or dyspnea. These symptoms can appear during or after exercise, or at rest, with variations in duration [39]. Considering the similarity with other CADs in symptom, the diagnosis of MVA is challenging [16]. Invasive examinations entail high risks and complications, long recovery time, and high costs. In addition, it is difficult to observe the hemodynamic changes in microvessels and myocardium. In contrast, the V/M ratio non-invasively derived from CCTA offers a comprehensive assessment of hemodynamic changes on macrovascular, microvascular, and myocardial levels. V/M ratio may offer a reliable alternative when invasive measurements like IMR are not available.

The impact of race on the incidence, severity, and prognosis of CAD, as well as cardiac geometry, is widely acknowledged [40]. One study indicated that the left ventricular mass of Asian ethnicities is significantly lower than that of Afro-Caribbeans and Europeans [41]. Compared with African American women and Caucasian men and women, African American men exhibit a larger left ventricular size and lower left ventricular systolic and diastolic function [14]. These studies underscore the ethnic differences in cardiac structure, with Asians exhibiting a lower left ventricular mass. Ihdayhid et al. compared the V/M ratio among 300 patients of Caucasian, South Asian, and East Asian descent presenting with chest pain, where East Asians exhibited the highest V/M value (29.2 mm^3/g) and total coronary lumen volume [15]. Grover et al. reported that V/M values in patients with PMVA were lower than those in the general population of Hispanic individuals [11]. In our study, the myocardial mass in the Chinese population was higher compared to Grover’s study, yet it remains within the normal range for left ventricular mass in the Chinese population [42]. Variations in myocardial mass between studies may be attributed to small sample sizes and differences in measurement techniques. The ethnic difference on V/M ratio and its diagnostic value for PMVA warrants further investigation.

This study did not incorporate acetylcholine challenge testing (AChT). The restricted adoption of AChT in China highlights systemic disparities between domestic clinical protocols and international standards. As outlined in the 2015 Chinese Expert Consensus on Coronary Spasm Syndrome [43], national guidelines recommend non-invasive provocation tests (e.g., cold pressor test) as first-line evaluations for vasospastic angina. This recommendation stems from the observations that East Asian populations exhibit significantly higher AChT positivity rates and complication rates [44, 45]. Despite the 2025 Consensus on Vasospastic Angina [46] recognizing AChT as the diagnostic gold standard, there are some technical barriers and the limitations in medical resources, including the dependence on imported cardiovascular-specific AChT reagents, the delayed domestic regulatory approval, and the gap in procedural standardization. Our protocol prioritized adenosine-mediated CFR and IMR assessments—an approach aligned with the tiered healthcare system in China. Future research should focus on establishing Chinese population-specific diagnostic thresholds through multicenter collaborations and validating non-invasive alternatives to address these limitations.

Second, the retrospective design of the study limits the ability to infer causal relationships and may introduce selection bias. In addition, due to the lack of detailed medication records, the potential effects of vasoactive agents and heart rate–modulating drugs on the V/M ratio could not be assessed. Inter-individual variability in CCTA image quality can influence the accuracy of morphometric measurements, as showcased in Fig. 8 where the anatomical complexity of LCX led to the high noises and procedural failures, limiting the number of cases available for analysis. Moreover, the absence of screening for other cardiac conditions that may lead to myocardial hypertrophy, such as valvular heart disease, hypertrophic cardiomyopathy, or hypertensive heart disease. This omission may have affected the interpretation of the relationship between the V/M ratio and PMVA, as these conditions can alter myocardial mass and coronary volume, potentially confounding the diagnostic specificity of the V/M ratio. Future studies should incorporate more comprehensive screening protocols to exclude such confounding factors and better define the independent diagnostic value of the V/M ratio in PMVA. Additionally, the V/M ratio could be integrated with other risk factors into a composite index to more comprehensively assess coronary microvascular function and predict the risk of PMVA.

##  Conclusion

The ratio of coronary artery lumen volume to myocardial mass is lower in Chinese patients with PMVA compared to other patients of angina without CMD. V/M based on CCTA may provide a non-invasive approach for diagnosing PMVA in East Asian population.

## Data Availability

The datasets used and analyzed during this study are available from the corresponding author on reasonable request.

## Abbreviations

CAD: Coronary artery disease
CMD: Coronary microvascular dysfunction
PMVA: Primary microvascular angina
CFR: Coronary flow reserve
FFR: Fractional flow reserve
IMR: Index of microcirculatory resistance
CCTA: Coronary computed tomography angiography
V/M: Ratio of coronary artery volume to myocardial mass
CT-FFR: CT-derived fractional flow reserve
LAD: Left anterior descending artery
LCX: Left circumflex artery
RCA: Right coronary artery
AHA: American heart association
ROC: Receiver operating characteristic
AUC: Area under the curve
BMI: Body mass index
CCS: Canadian cardiovascular society
NYHA: New York heart association
LVEF: Left ventricular ejection fraction
ALT: Alanine aminotransferase
AST: Aspartate aminotransferase
eGFR: Estimated glomerular filtration rate
HbA1c: Glycated hemoglobin
LDL-C: Low-density lipoprotein cholesterol
HDL-C: High-density lipoprotein cholesterol
UA: Uric acid
Cr: Creatinine
